# Isolated skin infiltration by a blastic plasmacytoid dendritic cell neoplasm

**DOI:** 10.1002/jha2.370

**Published:** 2022-01-10

**Authors:** Grégoire Stalder, Dina Milowich, Sabine Blum, Jacqueline Schoumans, Bettina Bisig, Olivier Spertini

**Affiliations:** ^1^ Service and Central Laboratory of Hematology Lausanne University Hospital and University of Lausanne Lausanne Switzerland; ^2^ Service of Histocytopathology Institut Central des Hôpitaux Hôpital du Valais Sion Switzerland; ^3^ Oncogenomic Laboratory Laboratory Department Lausanne University Hospital and University of Lausanne Lausanne Switzerland; ^4^ Institute of Pathology Lausanne University Hospital and University of Lausanne Lausanne Switzerland

A previously healthy 76‐year‐old woman presented with a slowly growing erythematous, painless papule on the left calf. The lesion progressively formed, within 3 months, a 5 × 3 cm indurated purplish nodule (Figure 1, panel A). Blood counts were normal and no sign of inflammatory diseases was found in the blood. A punch biopsy (Figure 1, panel D) of the calf lesion revealed diffuse dermic and hypodermic infiltration by medium‐ to large‐sized blasts, with convoluted nuclei and small nucleoli (Figure 1, panel E). The phenotype of the blasts was CD4+ (Figure 1, panel F), CD56+ (Figure 1, panel G), CD123+ (Figure 1, panel H), CD33+, TdT+/– (Figure 1, panel I), and MPO–, CD34–. T‐cell antigens, cytotoxic markers, and EBER in situ hybridization were negative. A diagnosis of blastic plasmacytoid dendritic cell neoplasm (BPDCN) was rendered. Next‐generation sequencing (NGS) on DNA extracted from the skin lesion showed mutations of *BRAF*, *NRAS*, and *TET2* at 37%, 5%, and 43% variant allele frequency (VAF), respectively. Bone marrow biopsy did not show neoplastic infiltration. Positron‐emission tomography revealed increased ^18^F‐fluorodeoxyglucose avidity in the calf lesion (Figure 1, panels B and C). BPDCN is a rare hematological malignancy with an overall incidence estimated to be 0.04 cases per 100,000 population per year. This disease most commonly manifests as cutaneous lesions with or without bone marrow involvement and leukemic dissemination. It is an aggressive tumor of very poor prognosis. The patient received two cycles of high‐dose cytarabine and cladribine chemotherapy, with macroscopic disappearance of the skin lesion after the first cycle and no evidence of mutations by NGS on the skin biopsy of the initial disease site after completion of the therapy. Treatment was followed by monthly cycles of 5‐azacytidine during 12 months. Thirty‐six months later, the BPDCN is still in complete remission.

**FIGURE 1:**
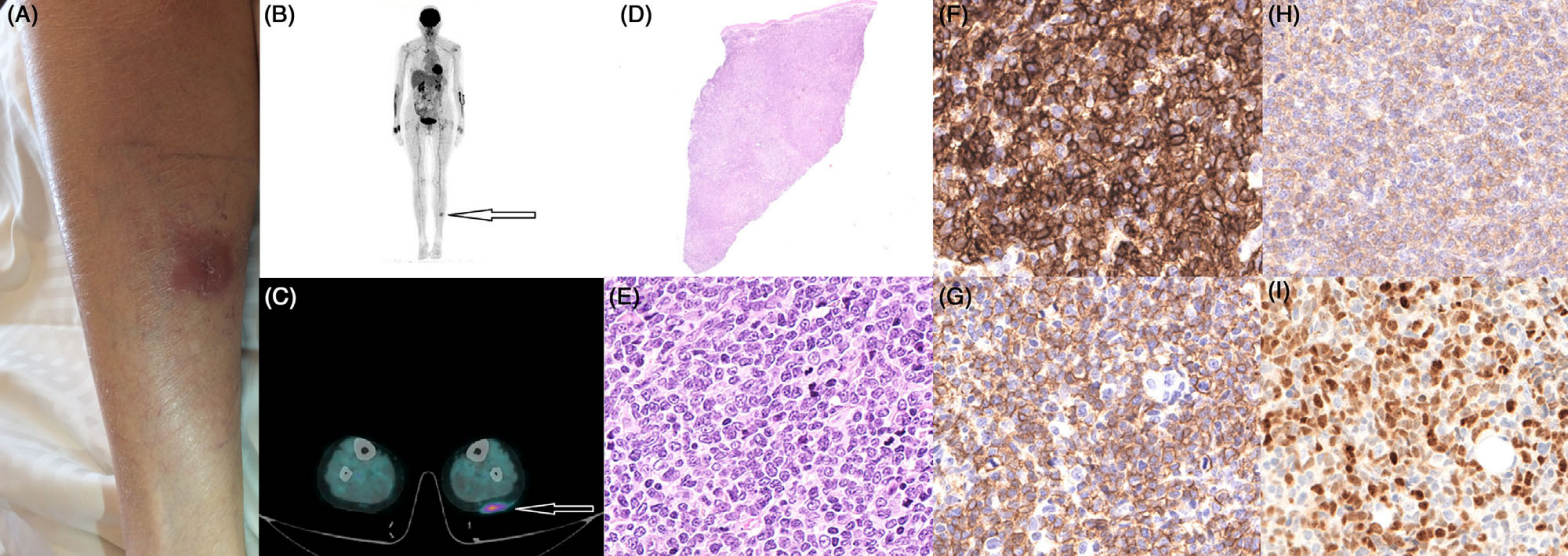
Photograph of the patient's left calf showing a 5 × 3 cm indurated purplish nodule (A). This lesion is hypermetabolic on PET‐CT, with no other lesions identified (B‐C). Biopsy of the nodule with hematoxylin‐eosin staining shows diffuse dermic and hypodermic infiltration by medium‐ to large‐sized blasts with convoluted nuclei and small nucleoli (D‐E). On immunohistochemistry, these blasts are CD4+ (F), CD56+ (G), CD123+ (H) TDT +/– (I).

## CONSENT STATEMENT

Signed informed consent was obtained from the patient included in this work.

